# Supplementary material for: Pharmacovigilance Strategies to Address Resistance to Antibiotics and Inappropriate Use—A Narrative Review
Source: Antibiotics (Basel). 2024 May 16;13(5):457. doi: 10.3390/antibiotics13050457 (PMC11117530; doi:10.3390/antibiotics13050457)
Supplement: Supplementary file 1 [file antibiotics-13-00457-s001.zip › antibiotics-3007773-supplementary.pdf]

Table S1. Studies that analysed signs and associations between adverse events and antibiotics in PV databases (2019-2023)

Author/Year	Type of Analysis	Period / Database	Major Results
Zou et al. 2023 [1]	Disproportionality	FAERS	There were 10 observed associations between antibiotic usage (AB) and the onset of seizures. Imipenem/cilastatin exhibited a greater risk.
Zhou et al. 2023 [2]	Disproportionality	2004 to 2022 FAERS	Thirty signs indicating severe cutaneous adverse reactions were observed, predominantly in female patients aged 18 to 65. Antibiotic-related reactions were notably common with: sulfonamides (ROR: 23.30), glycopeptides (ROR: 21.27), penicillins (ROR: 16.00), carbapenems (ROR: 10.46), and cephalosporins (ROR: 13.27).
Li et al. 2023 [3]	Disproportionality	2004 to 2022 FAERS	Findings indicate that metronidazole and vancomycin are associated with a greater risk of Clostridium difficile infection than other antibacterial agents, highlighting the imperative for further investigation."
Shao et al. 2023 [4]	Disproportionality	2013 to 2021 FAERS	The study found an association between linezolid and QT interval prolongation, especially in those with tuberculosis.
Liu et al. 2023 [5]	Disproportionality	2004 to 2021 FAERS	Finds identified potential risks for the development of hepatobiliary calculi associated with ceftriaxone treatment in male infants, children, and adolescents, as well as in female children and elderly women.
Seo and Kim 2023 [6]	Disproportionality	2004 to 2018 FAERS	Piperacillin-tazobactam is significantly associated with hypokalemia.
Chen et al. 2022 [7]	Disproportionality	2004 to 2021 FAERS	The results revealed the detection of 140 repetitive adverse events linked to daptomycin. Additionally, the study highlighted the identification of reports on rare adverse events, such as necrotizing fasciitis and compartment syndrome.
M Shaju et al. 2022 [8]	Disproportionality	FAERS / PV/Pharmacogenomics Insilico Pipeline (PHARMIP)	The study indicated antibiotics and other classes of medications associated with the induction of Red Man Syndrome, although the causal relationship has not been fully established.
Tang et al. 2022 [9]	Disproportionality	2013 to 2021 FAERS	The study associates the use of colistin with the occurrence of Stevens-Johnson syndrome and toxic epidermal necrolysis. Colistin was listed as a secondary suspect drug in 13 reports of such adverse events, representing 1.3% of the cases analyzed.

Heo et al. 2022 [10]	Disproportionality	2014 to 2018 Korea Adverse Events Reporting System	The authors identified nineteen adverse events associated with the use of doxycycline that were not previously included in the drug labels in six countries.
Recht et al. 2022 [11]	Análise de casos	FAERS / VigiBase	Three hundred and eighteen episodes of hemolytic anemia were reported in association with the use of nitrofurantoin. Among the episodes of hemolytic anemia, 42 (13%) occurred in individuals with confirmed or highly probable glucose-6-phosphate dehydrogenase deficiency.
Yamada et al. 2022 [12]	Disproportionality	2004 to 2020 JADER	The authors identified that patients with chronic kidney disease who were treated with ceftriaxone for more than 14 days, as well as women, are at risk of developing ceftriaxone-induced encephalopathy.
Kuula et al. 2022 [13]	Análise abrangente	2008 to 2019 Finnish Pharmaceutical Insurance Pool / Registro de Reações Adversas da Agência Finlandesa de Medicamentos.	It was estimated that 1,831,537 prescriptions of fluoroquinolones triggered 11,405 adverse drug reactions (ADRs) and 3,884 deaths during the period. The safety of fluoroquinolones is discussed along with their associations with serious adverse reactions, including recurrent Clostridioides difficile infections, cardiovascular toxicity, musculoskeletal disorders, renal and hepatic issues, as well as reactions involving the central nervous system.
Gatti et al. 2022 [14]	Disproportionality	FAERS / Eudravigilance	The authors observed a consistent signal of crystalline nephropathy associated with the use of amoxicillin, especially in France. Specific adverse events of interest included crystalluria, crystalline nephropathy, the presence of medication crystals in urine, crystalline urine, and the presence of crystals in urine.
Taher et al. 2022 [15]	Disproportionality	2010 to 2019 FAERS	The study identified moxifloxacin as the only quinolone with a positive signal of disproportionality for retinal detachment (RD). The positive signal indicates a potential association between the use of moxifloxacin and the risk of retinal detachment.
Mitsuboshi et al. 2022 [16]	Disproportionality	2004 to 2020 JADER	The authors suggest that the combination of oral vancomycin and intravenous piperacillin-tazobactam may increase the risk of renal injury.
Rey et al. 2022 [17]	Capture and recapture method	2019 French national pharmacovigilance database/cohort with	Antibiotics were the class most closely related to acute kidney injuries in hospitalized patients.

		patients from the Amiens Picardie University Hospital (Amiens, France).	
Jo et al. 2021 [18]	Case analysis	2010 to 2019 Korea Adverse Event Reporting System	The most common causative agents of fatal adverse events were antibacterials (20.3%), followed by antimycobacterials (5.4%), analgesics (4.0%), and contrast media (1.9%). Factors associated with fatal events included male sex, advanced age, polypharmacy, and the use of specific medications, including piperacillin/beta-lactamase inhibitor, cefotetan, ceftriaxone, combined antimycobacterial therapy, morphine, and iopromide.
Asai et al. 2021 [19]	Disproportionality	2004 to 2021 JADER	Anti-infectives associated with drug-induced thrombocytopenia include: ampicillin/sulbactam, ceftazidime, cefozopran, ciprofloxacin, fluconazole, fosfluconazole, linezolid, pazufloxacin, piperacillin/tazobactam, teicoplanin, sulfamethoxazole/trimethoprim, and voriconazole.
Largeau et al. 2021 [20]	Case analysis	2004 to 2019 French National Pharmacovigilance Database	The study identified a potential for amoxicillin to induce Drug Reaction with Eosinophilia and Systemic Symptoms (DRESS) syndrome, characterized by rash, fever, eosinophilia, and multi-organ dysfunction.
Gatti et al. 2021 [21]	Disproportionality	2014 to 2020 FAERS	The study investigated the adverse event profiles related to tedizolid and linezolid in post-marketing surveillance. The safety signal found is related to an increased reporting of hepatic failure with tedizolid.
Ge et al. 2021 [22]	Disproportionality	2015 to 2018 FAERS	The study raised concerns about carbapenem resistance in the United States and indicated that carbapenems are more likely to be associated with serious and fatal adverse events compared to other beta-lactam antibiotics.
Yamada et al. 2021 [23]	Disproportionality	2004 to 2020 JADER	This study suggests that daptomycin is associated with a risk of muscular toxicity and obesity, and concomitant use of statins may further increase this risk.
Nakao et al. 2021 [24]	Disproportionality	2004 to 2018 JADER	The results indicate that the risk of acute kidney injury associated with anti-infectives is higher in combination therapies (use of two or more anti-infectives) compared to monotherapy.

Nguyen et al. 2021 [25]	Disproportionality	2010 a 2016 Vietnam National FV Database	Reports of anaphylaxis occurred in 19.93% of cases involving beta-lactams. Additionally, the generation of anaphylaxis signal was found in specific subgroups, such as J01D (cephalosporins and carbapenems) and beta-lactamase-sensitive penicillins.
Rudolph et al. 2021 [26]	Disproportionality	Até 2017 VigiBase	Although the co-administration of tizanidine and ciprofloxacin is contraindicated, 91 ICSRs reporting this combination were identified. Adverse events mainly affect the nervous system and cardiac function.
Gatti et al. 2021 [27]	Disproportionality	2015 to 2020 FAERS	Agranulocytosis was identified as an unexpected adverse event for ceftolozane-tazobactam. Additionally, for ceftazidime-avibactam, acute pancreatitis was reported as a highly notified unexpected adverse event. Regarding neurological events, encephalopathy with antibiotics and alterations in mental status with ceftazidime-avibactam were highlighted as adverse events exhibiting significant disproportionality.
Lacroix et al. 2021 [28]	Disproportionality	1995 to 2017 French National Pharmacovigilance Database	Out of the 152 cases analyzed involving ceftriaxone, 112 patients were hospitalized or had prolonged hospitalization, 12 deaths were recorded, and 16 patients experienced life-threatening adverse events of the central nervous system.
Kan et al. 2021 [29]		1997 to 2017 FAERS / Japan Pharmaceutical Information Center	Signs of taste and smell disorders related to antibiotics were detected for amoxicillin, azithromycin, ciprofloxacin, clarithromycin, clindamycin, doxycycline, levofloxacin, and moxifloxacin.
Contejean et al. 2021 [30]	Disproportionality	1997 to 2019 VigiBase	The study found a disproportionality in reports of acute kidney injury in patients who received vancomycin in combination with piperacillin compared to vancomycin in other regimens.
Gatti et al. 2021 [31]	Disproportionality	até 2019 FAERS	Results suggest that linezolid is more likely to cause Serotonin Syndrome when co-administered with citalopram, escitalopram, and methadone, based on their pharmacological properties.
Akimoto et al. 2021 [32]	Disproportionality	2004 to 2020 (JADER)	Broad-spectrum antibiotics such as meropenem, tazobactam/piperacillin, and ceftriaxone were significantly associated with an increased risk of Drug-Induced Liver Injury. Meropenem had a higher risk in both databases.

Dai et al. 2020 [33]	Disproportionality	2013 to 2019 FAERS	The signal of lactic acidosis caused by linezolid was very high, and the incidence caused by other antibiotics was significantly different from that caused by linezolid. Elderly patients (⩾60 years) accounted for the largest proportion of cases, comprising 42.54% of the notifications. The event typically occurred two weeks after antibiotic administration.
Scavone et al. 2020 [34]	Disproportionality	Italian National Pharmacovigilance Network	Among the total adverse events reported for quinolones, 20.1% were associated with musculoskeletal, neurological, and psychiatric issues. Second-generation quinolones showed a lower likelihood of being linked to these problems compared to third-generation quinolones.
Villa Zapata et al. 2020 [35]	Disproportionality	2005 to 201 FAERS	A signal regarding the interaction between colchicine and clarithromycin was detected, underscoring the risks associated with this combination. These risks include serious complications such as diarrhea, pancytopenia, bone marrow failure, and even fatalities.
Zelmat et al. 2020 [36]	Desproporcionalildade	Up to 2017 VigiBase	The analysis revealed that sparfloxacin had the highest adjusted ROR for photosensitivity adverse events, followed by grepafloxacin, lomefloxacin, enoxacin, and fleroxacin. This finding suggests a correlation between the chemical structure of fluoroquinolones and the risk of photosensitivity, with a higher incidence of adverse effects reported for those containing a halogen in the 8th position.
Kennedy et al. 2020 [37]	Disproportionality	Up to 2017 FAERS	The analysis considered the concomitant use of antibiotics with glucose-lowering medications, including sulfonylureas and meglitinides. The results showed that the following antibiotics were significantly associated with hypoglycemia: cefditoren, tigecycline, ertapenem, and clarithromycin.
Timbrook et al. 2020 [38]	Disproportionality	2003 to 2018 FAERS	The results indicate that oxacillin showed a lower proportion of reports of acute renal failure and hypokalemia compared to nafcillin.
Patek et al. 2020 [39]	Disproportionality	2015 to 2017 FAERS	The results indicate the antibiotic classes related to acute kidney injury, in descending order: Colistin, Aminoglycosides, Vancomycin, Trimethoprim-Sulfamethoxazole, Penicillin combinations, Clindamycin, Cephalosporins, Daptomycin, Macrolides, Linezolid, Carbapenems, Metronidazole, Tetracyclines, Fluoroquinolones.

Bonaldo et al. 2019 [40]	Desproporcionalildade	VigiBase	The study analyzed ICSRs for macrolides and fluoroquinolones, comparing them to amoxicillin. The safety signal identified is the more frequent association of macrolides with atrial fibrillation and ventricular fibrillation compared to fluoroquinolones.
Thornhill et al. 2019 [41]	Disproportionality	2010 to 2017 NHS Digital Prescribing	It found variations in the risks of AEs among different antibiotics prescribed by dentists, highlighting the high rate of both fatal and non-fatal AEs associated with the use of clindamycin.
Orion et al. 2019 [42]	Disproportionality	Up to 2017 VigiBase	The study identified an association between Kounis syndrome and the use of antibiotics and analgesics, with amoxicillin/clavulanate and ibuprofen mentioned as the substances most frequently suspected.
Sommet et al. 2019 [43]	Desproporcionalildade	1972 to 2017 Vigibase	The results showed an increased risk of aortic aneurysms and dissections associated with the use of fluoroquinolones. Specifically, the risk was significantly higher with the use of levofloxacin compared to other fluoroquinolones.
Lacroix et al. 2019 [44]	Análise de casos	1987 to 2017 French National Pharmacovigilance Database	The study found associations of cephalosporins with severe Adverse Events (AEs) in the Central Nervous System, including encephalopathy, confusion, seizures, myoclonus, status epilepticus, coma, and hallucinations.
Chandler et al. 2019 [45]	Desproporcionalildade	Up to 2018 VigiBase	A higher disproportionality was found in reports of drug-induced aseptic meningitis related to amoxicillin/clavulanic acid in the male subgroup.
Teng et al. 2019 [46]	Desproporcionalildade	2015 to 2017 FAERS	A significant association was identified between the use of different classes of antibiotics and Clostridium difficile infection. Lincosamides, such as clindamycin, showed the highest reporting rate among the evaluated antibiotic classes.
Morales et al. 2019 [47]	Nested case-control study	The UK Health Improvement Network primary care database	A tendon rupture risk was associated with fluoroquinolone use; however, it was observed that such a signal depends on timing, dosage, and concurrent exposure to corticosteroids. These signals were more frequently observed in elderly patients.
Morales et al. 2019 [48]	Nested case-control study	1999 to 2015 The Health Improvement Network database	Oral fluoroquinolone exposure was linked to a higher relative incidence of peripheral neuropathy compared to non-exposure. No

			significant increased risk was observed with oral amoxicillin-clavulanate exposure.
Teng et al. 2019 [49]	Disproportionality	2015 to 2017 FAERS	The study confirmed evidence of the association of Torsades de Pointes/prolongation of the QT interval (TdP/QTP) with macrolides, linezolid, imipenem-cilastatin, fluoroquinolones, penicillin combinations, and ceftriaxone. An association was also made between the use of amikacin and the TdP/QTP signal.

Abbreviations: FAERS: FDA Adverse Event Reporting System; **AB:** Antibiotic; **PV:** Pharmacovigilance; **JADER:** Japanese Adverse Drug Event Report; **ICSR:** Individual Case Safety Report; **ROR:** Reporting Odds Ratio.

References

1. Zou, D.; Zhang, R.; Yu, L.; Hu, T.; Wu, B. Seizures Associated with Antibiotics: A Real-World Disproportionality Analysis of FAERS Database. *Expert Opin Drug Saf* **2023**, *22*, 1143–1148, doi:10.1080/14740338.2023.2234825.
2. Zhou, L.; Yang, J.; Xiao, M.; Shan, H.; Liu, M.; Lu, Y.; Zou, Y.; Wu, B. Severe Cutaneous Adverse Reactions Due to Antibiotics Therapy: A Pharmacovigilance Analysis of FDA Adverse Event Reporting System Events. *Expert Opin Drug Saf* **2023**, 1–8, doi:10.1080/14740338.2023.2278685.
3. Li, D.; Song, Y.; Bai, Z.; Xi, X.; Liu, F.; Zhang, Y.; Qin, C.; Du, D.; Du, Q.; Liu, S. Real-World Data in Pharmacovigilance Database Provides a New Perspective for Understanding the Risk of Clostridium Difficile Infection Associated with Antibacterial Drug Exposure. *Antibiotics (Basel)* **2023**, *12*, doi:10.3390/antibiotics12071109.
4. Shao, H.; Shi, D.; Dai, Y. Linezolid and the Risk of QT Interval Prolongation: A Pharmacovigilance Study of the Food and Drug Administration Adverse Event Reporting System. *Br J Clin Pharmacol* **2023**, *89*, 1386–1392, doi:10.1111/bcp.15587.
5. Liu, X.; Xu, Z.; Ma, J.; Zhang, A.; Li, Z.; Qi, G.; Li, Z.; Wei, F.; Zhong, L. Hepatobiliary Calculi Associated with Ceftriaxone Treatment: An Analysis of FAERS Data from 2004 to 2021. *J Infect Chemother* **2023**, *29*, 136–142, doi:10.1016/j.jiac.2022.10.006.
6. Seo, H.; Kim, E. Electrolyte Disorders Associated with Piperacillin/Tazobactam: A Pharmacovigilance Study Using the FAERS Database. *Antibiotics (Basel)* **2023**, *12*, doi:10.3390/antibiotics12020240.
7. Chen, J.-J.; Huo, X.-C.; Wang, S.-X.; Wang, F.; Zhao, Q. Data Mining for Adverse Drug Reaction Signals of Daptomycin Based on Real-World Data: A Disproportionality Analysis of the US Food and Drug Administration Adverse Event Reporting System. *Int J Clin Pharm* **2022**, *44*, 1351–1360, doi:10.1007/s11096-022-01472-x.
8. M Shaju, A.; Panicker, N.; Chandni, V.; Lakshmi Prasanna, V.M.; Nair, G.; Subeesh, V. Drugs-Associated with Red Man Syndrome: An Integrative Approach Using Disproportionality Analysis and Pharmip. *J Clin Pharm Ther* **2022**, *47*, 1650–1658, doi:10.1111/jcpt.13716.
9. Tang, R.; Lopes, V.L.; Caffrey, A.R. Colistin-Associated Stevens-Johnson Syndrome and Toxic Epidermal Necrolysis Reactions: A Retrospective Case-Non-Case Pharmacovigilance Study. *Expert Opin Drug Saf* **2022**, *21*, 1121–1126, doi:10.1080/14740338.2022.2045945.
10. Heo, J.Y.; Cho, M.K.; Kim, S. Data Mining for Detecting Signals of Adverse Drug Reaction of Doxycycline Using the Korea Adverse Event Reporting System Database. *J Dermatolog Treat* **2022**, *33*, 2192–2197, doi:10.1080/09546634.2021.1937480.

11. Recht, J.; Chansamouth, V.; White, N.J.; Ashley, E.A. Nitrofurantoin and Glucose-6-Phosphate Dehydrogenase Deficiency: A Safety Review. *JAC Antimicrob Resist* **2022**, *4*, dlac045, doi:10.1093/jacamr/dlac045.
12. Yamada, T.; Mitsuboshi, S.; Suzuki, K.; Nishihara, M.; Neo, M. Analysis of the Frequency of Ceftriaxone-Induced Encephalopathy Using the Japanese Adverse Drug Event Report Database. *Int J Clin Pharm* **2022**, *44*, 1067–1071, doi:10.1007/s11096-022-01406-7.
13. Kuula, L.S.M.; Backman, J.T.; Blom, M.L. Healthcare Costs and Mortality Associated with Serious Fluoroquinolone-Related Adverse Reactions. *Pharmacol Res Perspect* **2022**, *10*, e00931, doi:10.1002/prp2.931.
14. Gatti, M.; Fusaroli, M.; Raschi, E.; Capelli, I.; Poluzzi, E.; De Ponti, F. Crystal Nephropathy and Amoxicillin: Insights from International Spontaneous Reporting Systems. *J Nephrol* **2022**, *35*, 1017–1027, doi:10.1007/s40620-021-01191-y.
15. Taher, M.K.; Alami, A.; Gravel, C.A.; Tsui, D.; Bjerre, L.M.; Momoli, F.; Mattison, D.; Krewski, D. Systemic Quinolones and Risk of Retinal Detachment I: Analysis of Data from the US FDA Adverse Event Reporting System. *Expert Opin Drug Saf* **2022**, *21*, 269–276, doi:10.1080/14740338.2022.1993187.
16. Mitsuboshi, S.; Katagiri, H. Risk of Kidney Injury in Patients on Concomitant Oral Vancomycin and Piperacillin-Tazobactam: Analysis of the Pharmacovigilance Database in Japan. *Basic Clin Pharmacol Toxicol* **2022**, *130*, 208–212, doi:10.1111/bcpt.13689.
17. Rey, A.; Gras, V.; Moragny, J.; Choukroun, G.; Masmoudi, K.; Liabeuf, S. Use of the Capture-Recapture Method to Estimate the Frequency of Community- and Hospital-Acquired Drug-Induced Acute Kidney Injuries in French Databases. *Front Pharmacol* **2022**, *13*, 899164, doi:10.3389/fphar.2022.899164.
18. Jo, H.-G.; Jeong, K.; Ryu, J.-Y.; Park, S.; Choi, Y.-S.; Kwack, W.-G.; Choi, Y.-J.; Chung, E.-K. Fatal Events Associated with Adverse Drug Reactions in the Korean National Pharmacovigilance Database. *J Pers Med* **2021**, *12*, doi:10.3390/jpm12010005.
19. Asai, Y.; Yamamoto, T.; Abe, Y. Evaluation of the Expression Profile of Antibiotic-Induced Thrombocytopenia Using the Japanese Adverse Drug Event Report Database. *Int J Toxicol* **2021**, *40*, 542–550, doi:10.1177/10915818211048151.
20. Largeau, B.; Agier, M.-S.; Beau-Salinas, F.; Pariente, A.; Maruani, A.; Vial, T.; Jonville-Béra, A.-P. Specific Features of Amoxicillin-Associated Drug Reaction with Eosinophilia and Systemic Symptoms Syndrome: A Nationwide Study. *J Eur Acad Dermatol Venereol* **2021**, *35*, 2415–2420, doi:10.1111/jdv.17631.
21. Gatti, M.; Fusaroli, M.; Raschi, E.; Moretti, U.; Poluzzi, E.; De Ponti, F. Serious Adverse Events with Tedizolid and Linezolid: Pharmacovigilance Insights through the FDA Adverse Event Reporting System. *Expert Opin Drug Saf* **2021**, *20*, 1421–1431, doi:10.1080/14740338.2021.1956461.
22. Ge, W.; Hu, H.; Li, C.; Wang, L.; Xia, J. Safety Profile of Carbapenems: Data Mining of the FDA Adverse Events Reporting System. *Int J Clin Pharmacol Ther* **2021**, *59*, 594–602, doi:10.5414/CP203811.
23. Yamada, T.; Mitsuboshi, S.; Suzuki, K.; Nishihara, M.; Uchiyama, K. Risk of Muscle Toxicity Events for Daptomycin with and without Statins: Analysis of the Japanese Adverse Event Report Database. *Basic Clin Pharmacol Toxicol* **2021**, *129*, 268–272, doi:10.1111/bcpt.13618.
24. Nakao, S.; Hasegawa, S.; Umetsu, R.; Shimada, K.; Mukai, R.; Tanaka, M.; Matsumoto, K.; Yoshida, Y.; Inoue, M.; Satake, R.; et al. Pharmacovigilance Study of Anti-Infective-Related Acute Kidney Injury Using the Japanese Adverse Drug Event Report Database. *BMC Pharmacol Toxicol* **2021**, *22*, 47, doi:10.1186/s40360-021-00513-x.
25. Nguyen, K.-D.; Vu, D.-H.; Nguyen, H.-A.; Dao, V.-T.; Montastruc, J.-L.; Bagheri, H. Risk Comparison of Beta-Lactam-Induced Anaphylaxis: Therapeutic Stratification Analysis in a Vietnamese Pharmacovigilance Database. *J Clin Pharm Ther* **2021**, *46*, 950–956, doi:10.1111/jcpt.13376.

26. Rudolph, A.; Dahmke, H.; Kupferschmidt, H.; Burden, A.; Weiler, S. Coadministration of Tizanidine and Ciprofloxacin: A Retrospective Analysis of the WHO Pharmacovigilance Database. *Eur J Clin Pharmacol* **2021**, *77*, 895–902, doi:10.1007/s00228-020-02981-2.
27. Gatti, M.; Raschi, E.; De Ponti, F. Serious Adverse Events with Novel Beta-Lactam/Beta-Lactamase Inhibitor Combinations: A Large-Scale Pharmacovigilance Analysis. *Eur J Clin Microbiol Infect Dis* **2021**, *40*, 1169–1176, doi:10.1007/s10096-020-04149-3.
28. Lacroix, C.; Bera-Jonville, A.-P.; Montastruc, F.; Velly, L.; Micallef, J.; Guilhaumou, R. Serious Neurological Adverse Events of Ceftriaxone. *Antibiotics (Basel)* **2021**, *10*, doi:10.3390/antibiotics10050540.
29. Kan, Y.; Nagai, J.; Uesawa, Y. Evaluation of Antibiotic-Induced Taste and Smell Disorders Using the FDA Adverse Event Reporting System Database. *Sci Rep* **2021**, *11*, 9625, doi:10.1038/s41598-021-88958-2.
30. Contejean, A.; Tisseyre, M.; Canouï, E.; Treluyer, J.-M.; Kerneis, S.; Chouchana, L. Combination of Vancomycin plus Piperacillin and Risk of Acute Kidney Injury: A Worldwide Pharmacovigilance Database Analysis. *J Antimicrob Chemother* **2021**, *76*, 1311–1314, doi:10.1093/jac/dkab003.
31. Gatti, M.; Raschi, E.; De Ponti, F. Serotonin Syndrome by Drug Interactions with Linezolid: Clues from Pharmacovigilance-Pharmacokinetic/Pharmacodynamic Analysis. *Eur J Clin Pharmacol* **2021**, *77*, 233–239, doi:10.1007/s00228-020-02990-1.
32. Akimoto, H.; Nagashima, T.; Minagawa, K.; Hayakawa, T.; Takahashi, Y.; Asai, S. Signal Detection of Potential Hepatotoxic Drugs: Case-Control Study Using Both a Spontaneous Reporting System and Electronic Medical Records. *Biol Pharm Bull* **2021**, *44*, 1514–1523, doi:10.1248/bpb.b21-00407.
33. Dai, Y.; Wang, Y.; Zeng, Y.; Zhang, C.; Zhou, Z.; Shi, D. Linezolid and the Risk of Lactic Acidosis: Data Mining and Analysis of the FDA Adverse Event Reporting System. *J Clin Pharm Ther* **2020**, *45*, 1422–1426, doi:10.1111/jcpt.13245.
34. Scavone, C.; Mascolo, A.; Ruggiero, R.; Sportiello, L.; Rafaniello, C.; Berrino, L.; Capuano, A. Quinolones-Induced Musculoskeletal, Neurological, and Psychiatric ADRs: A Pharmacovigilance Study Based on Data From the Italian Spontaneous Reporting System. *Front Pharmacol* **2020**, *11*, 428, doi:10.3389/fphar.2020.00428.
35. Villa Zapata, L.; Hansten, P.D.; Horn, J.R.; Boyce, R.D.; Gephart, S.; Subbian, V.; Romero, A.; Malone, D.C. Evidence of Clinically Meaningful Drug-Drug Interaction With Concomitant Use of Colchicine and Clarithromycin. *Drug Saf* **2020**, *43*, 661–668, doi:10.1007/s40264-020-00930-7.
36. Zelmat, Y.; Rousseau, V.; Chebane, L.; Montastruc, J.-L.; Bagheri, H.; Sommet, A. Fluoroquinolone-Induced Photosensitivity: A Chemical Fragment-Based Approach by a Case/Non-Case Study in VigiBase(®). *Drug Saf* **2020**, *43*, 561–566, doi:10.1007/s40264-020-00917-4.
37. Kennedy, K.E.; Teng, C.; Patek, T.M.; Frei, C.R. Hypoglycemia Associated with Antibiotics Alone and in Combination with Sulfonylureas and Meglitinides: An Epidemiologic Surveillance Study of the FDA Adverse Event Reporting System (FAERS). *Drug Saf* **2020**, *43*, 363–369, doi:10.1007/s40264-019-00901-7.
38. Timbrook, T.T.; McKay, L.; Sutton, J.D.; Spivak, E.S. Disproportionality Analysis of Safety with Nafcillin and Oxacillin with the FDA Adverse Event Reporting System (FAERS). *Antimicrob Agents Chemother* **2020**, *64*, doi:10.1128/AAC.01818-19.
39. Patek, T.M.; Teng, C.; Kennedy, K.E.; Alvarez, C.A.; Frei, C.R. Comparing Acute Kidney Injury Reports Among Antibiotics: A Pharmacovigilance Study of the FDA Adverse Event Reporting System (FAERS). *Drug Saf* **2020**, *43*, 17–22, doi:10.1007/s40264-019-00873-8.
40. Bonaldo, G.; Andriani, L.A.; D'Annibali, O.; Motola, D.; Vaccheri, A. Cardiovascular Safety of Macrolide and Fluoroquinolone Antibiotics: An Analysis of the WHO Database of

Adverse Drug Reactions. *Pharmacoepidemiol Drug Saf* **2019**, *28*, 1457–1463, doi:10.1002/pds.4873.
41. Thornhill, M.H.; Dayer, M.J.; Durkin, M.J.; Lockhart, P.B.; Baddour, L.M. Risk of Adverse Reactions to Oral Antibiotics Prescribed by Dentists. *J Dent Res* **2019**, *98*, 1081–1087, doi:10.1177/0022034519863645.
42. Orion, K.; Mack, J.; Kullak-Ublick, G.A.; Weiler, S. Kounis Syndrome: A Retrospective Analysis of Individual Case Safety Reports from the International WHO Database in Pharmacovigilance. *Int J Clin Pharmacol Ther* **2019**, *57*, 240–248, doi:10.5414/CP203344.
43. Sommet, A.; Bénévent, J.; Rousseau, V.; Chebane, L.; Douros, A.; Montastruc, J.-L.; Montastruc, F. What Fluoroquinolones Have the Highest Risk of Aortic Aneurysm? A Case/Non-Case Study in VigiBase®. *J Gen Intern Med* **2019**, *34*, 502–503, doi:10.1007/s11606-018-4774-2.
44. Lacroix, C.; Kheloufi, F.; Montastruc, F.; Bennis, Y.; Pizzoglio, V.; Micallef, J. Serious Central Nervous System Side Effects of Cephalosporins: A National Analysis of Serious Reports Registered in the French Pharmacovigilance Database. *J Neurol Sci* **2019**, *398*, 196–201, doi:10.1016/j.jns.2019.01.018.
45. Chandler, R.E. Increased Risk for Aseptic Meningitis after Amoxicillin or Amoxicillin-Clavulanic Acid in Males: A Signal Revealed by Subset Disproportionality Analysis within a Global Database of Suspected Adverse Drug Reactions. *Pharmacoepidemiol Drug Saf* **2019**, *28*, 389–395, doi:10.1002/pds.4707.
46. Teng, C.; Reveles, K.R.; Obodozie-Ofoegbu, O.O.; Frei, C.R. Clostridium Difficile Infection Risk with Important Antibiotic Classes: An Analysis of the FDA Adverse Event Reporting System. *Int J Med Sci* **2019**, *16*, 630–635, doi:10.7150/ijms.30739.
47. Morales, D.R.; Slattery, J.; Pacurariu, A.; Pinheiro, L.; McGettigan, P.; Kurz, X. Relative and Absolute Risk of Tendon Rupture with Fluoroquinolone and Concomitant Fluoroquinolone/Corticosteroid Therapy: Population-Based Nested Case-Control Study. *Clin Drug Investig* **2019**, *39*, 205–213, doi:10.1007/s40261-018-0729-y.
48. Morales, D.; Pacurariu, A.; Slattery, J.; Pinheiro, L.; McGettigan, P.; Kurz, X. Association Between Peripheral Neuropathy and Exposure to Oral Fluoroquinolone or Amoxicillin-Clavulanate Therapy. *JAMA Neurol* **2019**, *76*, 827–833, doi:10.1001/jamaneurol.2019.0887.
49. Teng, C.; Walter, E.A.; Gaspar, D.K.S.; Obodozie-Ofoegbu, O.O.; Frei, C.R. Torsades de Pointes and QT Prolongation Associations with Antibiotics: A Pharmacovigilance Study of the FDA Adverse Event Reporting System. *Int J Med Sci* **2019**, *16*, 1018–1022, doi:10.7150/ijms.34141.
